# Uniparental Isodisomy of Chromosome 1 Unmasking an Autosomal Recessive 3-Beta Hydroxysteroid Dehydrogenase Type II-Related Congenital Adrenal Hyperplasia

**DOI:** 10.4274/jcrpe.3680

**Published:** 2017-03-01

**Authors:** Karin Panzer, Osayame A. Ekhaguere, Benjamin Darbro, Jennifer Cook, Oleg A. Shchelochkov

**Affiliations:** 1 University of Iowa Hospitals and Clinics, Stead Department of Pediatrics, Iowa, USA; 2 The Children’s Hospital of Philadelphia, Division of Neonatal and Perinatal Medicine, Philadelphia, USA; 3 Blank Children’s Hospital, Department of Pediatric Endocrinology, Iowa, USA; 4 Current Institution: National Human Genome Research Institute, Bethesda, Maryland, USA

**Keywords:** Steroid 3β-HSD2 deficiency, HSD3B2 gene, uniparental isodisomy

## Abstract

Steroid 3-beta hydroxysteroid dehydrogenase type II (3β-HSD2) deficiency is a rare autosomal recessive form of congenital adrenal hyperplasia (CAH). We report the genetic basis of 3β-HSD2 deficiency arising from uniparental isodisomy (UPD) of chromosome 1. We describe a term undervirilized male whose newborn screen indicated borderline CAH. The patient presented on the 7^th^ day of life in salt-wasting adrenal crisis. Steroid hormone testing revealed a complex pattern suggestive of 3β-HSD deficiency. Chromosomal microarray and single nucleotide polymorphism analysis revealed complete UPD of chromosome 1. Sanger sequencing of *HSD3B2* revealed a previously described missense mutation, c.424G>A (p.E142K) in homozygous state, thus confirming the diagnosis of 3β-HSD2 deficiency. We provide evidence of the existence of an uncommon mechanism for *HSD3B2* gene-related CAH arising from UPD of chromosome 1.

**WHAT IS ALREADY KNOWN ON THIS TOPIC?**

3-beta hydroxysteroid dehydrogenase type II (3β-HSD2) deficiency is a rare form of congenital adrenal hyperplasia (CAH) that is inherited in an autosomal recessive manner, typically with one gene variant inherited from each parent. Uniparental isodisomy (UPD) as the genetic basis of CAH has been reported in other forms of CAH.

**WHAT THIS STUDY ADDS?**

The first reported case of 3β-HSD2 deficiency arising from UPD of chromosome 1.

## INTRODUCTION

Deficiency of 3-beta hydroxysteroid dehydrogenase type II (3β-HSD2) is a rare autosomal recessive form of congenital adrenal hyperplasia (CAH). In humans, 3β-HSD2 is predominantly expressed in the adrenal glands and gonads (1) and its encoding gene, *HSD3B2* (MIM*613890), is located on chromosome 1 (2,3). The enzyme 3β-HSD2 oxidizes and isomerizes Δ^5^-steroids, namely, pregnenolone, 17-hydroxy-pregnenolone (17-OH Preg) and dehydroepiandrosterone into corresponding Δ^4^-ketosteroids (4). Deficiency of the enzyme impacts the steroid hormone pathway by disrupting the biosynthesis of mineralocorticoid, corticosteroid, and sex hormones. Typically, 3β-HSD2 enzyme deficiency results in cortisol deficiency, salt wasting, and male undervirilization. However, depending on the degree of enzyme deficiency and activity of the 3-beta hydroxysteroid dehydrogenase type I (3β-HSD1) enzyme, a similar enzyme produced in the skin and placenta, milder presentation may occur (5). This variability in clinical presentation should prompt clinicians to seek clarification of the diagnosis using molecular methods.

Increased utilization of single nucleotide polymorphism (SNP) array in clinical practice has led to the recognition of uniparental isodisomy (UPD) that either disrupts imprinting patterns or unmasks autosomal recessive alleles (6). Reports on UPD as an underlying molecular mechanism of CAH are scarce (7). There are reported cases of UPD involving the two most common forms of CAH, steroid 21-hydroxylase (8) and 11-β-hydroxylase deficiency (9). Here, we report the first patient affected by *HSD3B2*-related CAH uncovered by UPD of chromosome 1. In general, UPD of chromosome 1 has been infrequently reported in other disease conditions (10).

## CASE REPORT

Our patient is a 7-day-old male infant born at 37 weeks 5 days gestation. He was taken to his primary care physician with complaints of poor weight gain and vomiting and was referred to our hospital.

The infant was the product of an uncomplicated pregnancy, born to Caucasian parents. His birth length was 50.8 cm (50th percentile), birth weight 3400 grams (50^th^ percentile), and head circumference 38 cm (75^th^ percentile). Physical examination was significant for hypotonia, non-specific dysmorphic facial findings including slight frontal bossing, hypotelorism, low nasal bridge and anteverted nares. Also noted was perineal hypospadias, bifid scrotum, penile chordee, and descended testes bilaterally. As part of the work-up for his nonspecific dysmorphic facies and hypotonia, an oligonucleotide microarray and a SNP analysis was sent. Also, at newborn screening, the patient was found to have a 17-hydroxyprogesterone (17-OHP) level of 33.2 ng/mL, borderline for his birthweight (normal limits <30, borderline is 30-74, and presumptive positive is ≥75).

Laboratory evaluation revealed hyponatremia with a serum sodium level of 131 mEq/L (reference 135-145 mEq/L) with concurrent hyperkalemia and a potassium level of 7.9 mEq/L (reference 3.5-4.5 mEq/L). He was admitted to the pediatric intensive care unit and started on hydrocortisone, fludrocortisone, and sodium chloride supplementation. Steroid hormone testing obtained before treatment revealed a complex pattern suggestive of 3β-HSD deficiency. Steroid hormone determinations revealed the following abnormal values: 17-OH Preg 119.0 nmol/L (reference 0.3-26.2 nmol/L), 17-OHP 16.9 nmol/L (reference 1.3-6.4 nmol/L), dehydroepiandrosterone 95.4 nmol/L (reference 1.7-26.4 nmol/L), progesterone 1.8 nmol/L (reference <0.3-0.5 nmol/L), cortisol 1462.3 nmol/L (reference 77.3-303.5 nmol/L), 11-deoxycortisol 9.9 nmol/L (reference ≤5.9 nmol/L), 11-desoxycorticosterone 0.2 nmol/L (reference 1.0-2.7 nmol/L), and androstenedione 9.7 nmol/L (reference <1.8 nmol/L). Other laboratory results included: adrenocorticotropic hormone 8.4 pmol/L (reference 1.3-10.6 pmol/L) and testosterone 1.6 nmol/L (reference 0.7-1.7 nmol/L). Additional studies included chromosomal analysis, chromosomal microarray, and fluorescent in situ hybridization analysis (FISH) of sex-determining region Y (SRY).

The patient recovered from his acute illness, was discharged home on steroids and electrolyte replacements. He underwent urologic surgery to correct his urogenital anomalies.

### Genetic Analysis

Chromosomal karyotype analysis was performed per standard technique. FISH studies were completed with Vysis SRY probe using standard technique. Chromosomal oligonucleotide microarray and SNP analysis was done using an Affymetrix CytoScanHD hg19 (NCBI build 37) whole genome array consisting of 1.9 million non-polymorphic markers and 750,000 SNP probes, with an average probe spacing of about 1.2 kb. Data were extracted and processed using Affymetrix ChAS software (Affymetrix, version 1.2.2) and Nexus Copy Number (BioDiscovery, version 7) software.

**Figure 1:**
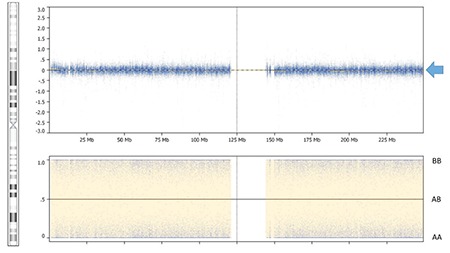
Uniparental isodisomy of chromosome 1. The figure demonstrates a normal copy number of chromosome 1 represented by probes in the top panel (blue arrow) averaging log_2_R=0 in the absence of AB alleles shown in the bottom panel

Chromosomal analysis revealed normal male complement, 46,XY. SRY was present by FISH study. Chromosomal oligonucleotide microarray and SNP analysis revealed complete UPD of chromosome 1 (Figure 1), a 264 kb deletion of 11q14.1, and a 517 kb duplication of 17p13.2. The microdeletion of 11q14.1 and microduplication of 17p13.2 did not involve genes associated with known human disorders. Since UPD can clinically unmask mutations implicated in autosomal recessive disorders, gene content of chromosome 1 was reviewed for candidates associated with autosomal recessive syndromic ambiguous genitalia. Several genes met this criterion, including HSD3B2. Sanger sequencing of HSD3B2 revealed a previously described missense mutation, c.424G>A (p.E142K) in a homozygous state, thus confirming the diagnosis of 3β-HSD2. Parental samples were not available for our review to establish the origin of mutation and the parental origin of UPD.

## DISCUSSION

This is the first report to demonstrate the role of UPD on chromosome 1 as the molecular basis for the rare *HSD3B2*-related CAH, unmasking a known homozygous missense mutation, c.424G>A (p.E142K). Mutation c.424G>A (p.E142K) was uncovered as an incidental finding during a work-up for non-endocrine indication of dysmorphic appearance and hypotonia. This mutation has previously been implicated in the development of classic and non-classic cases of *HSD3B2*-related CAH. Simard et al (11) reported an undervirilized male with salt-wasting CAH caused by compound heterozygosity of missense mutation p.E142K and a nonsense mutation p.W171X in HSD3B2. Pang et al (12) reported a female patient with non-classic presentation of 3β-HSD deficiency causing premature sexual hair growth and mild growth acceleration in childhood. Molecular analysis revealed compound heterozygous c.1022C>T (p.Pro341Leu) and c.424G>A (p.E142K) mutations in *HSD3B2*.

Steroid hormone testing in our patient revealed a complex pattern of abnormalities. In addition to an elevated 17-OH Preg (a 4.5-fold increase above the upper normal range) and dehydroepiandrosterone (a 3.6-fold increase above the upper normal range), the patient’s profile revealed elevations of Δ^4^-ketosteroids and their products (progesterone, 17-OHP, androstenedione, and cortisol). ACTH and testosterone were in the normal range. Elevations of Δ^4^-ketosteroids and their downstream metabolites in patients with 3β-HSD2 deficiency have been reported in the literature (1). Increased levels of 17-OHP, androstenedione, and testosterone are thought to occur due to preserved activity of 3β-HSD1 encoded by a paralogue gene *HSD3B1* expressed postnatally in skin and placenta (13). Therefore, it has been suggested that an elevated ratio of Δ^5^/Δ^4^ -steroids could be a more informative biomarker to ascertain 3β-HSD2 deficiency (1).

Genetic counseling for *HSD3B2*-related CAH involves a discussion of autosomal recessive inheritance and a 25% chance for parents to have another child with CAH. However, the recurrence risk for CAH arising from UPD of a complete chromosome is considered much smaller owing to the mechanism underlying isodisomy formation. UPD in humans, as first described by Engel (14) in 1980, is the presence of a chromosome pair or portions of a chromosome pair (15) that originate from a single parent, thus designated maternal or paternal. Mechanistic explanations of UPD involve gamete complementation, trisomic rescue, monosomic rescue, or post-fertilization mitotic error (16,17). The first two explanations would result in uniparental heterodisomy, while monosomic rescue and mitotic error and rescue may result in UPD. In all these cases, the original abnormality is thought to be a sporadic event without a significantly increased risk of recurrence, and parental chromosomal analysis would reveal normal results. Rarer mechanisms to generate complete UPD include correction of interchange trisomy or monosomy (in connection with a Robertsonian or reciprocal translocation), isochromosome formation, and correction of imbalance due to extra structurally abnormal chromosomes. In these rarer mechanisms, the parental karyotype should reveal a predisposing balanced or unbalanced chromosomal complement, and as such, may have an increased risk for recurrence. As parental samples were not unavailable, we could not establish the parental origin of UPD in this patient. However, the complete loss of heterozygosity of chromosome 1 in this patient suggests that monosomic rescue or mitotic error were the two most likely mechanisms of the observed UPD. In practice, since both of these mechanisms require the sequential occurrence of two abnormal events, UPD is usually a sporadic event with low risk of recurrence (18).

Follow-up and management of patients with HSD3B2-related CAH have been well established (19). However, in formulating the management of autosomal recessive disorders unmasked by UPD, one needs to consider the possibility of other clinically relevant autosomal recessive alleles that could be revealed through the UPD-mediated loss of heterozygosity (20). Additional copy number variants identified by microarray may cause additive effects or modify the clinical presentation for this patient over time. We speculate this mechanism may have contributed to the non-specific dysmorphic features and hypotonia in this patient. Finally, to date, most phenotypes associated with UPD of chromosome 1 have been linked to the autosomal recessive disorders without evidence for the existence of a possible imprinting disorder.

In conclusion, with this case report, we provide evidence for the existence of an uncommon mechanism of HSD3B2-related CAH arising from UPD of chromosome 1 that required the use of SNP-based array in the molecular evaluation of the patient, both from a diagnostic standpoint and recurrence risk assessment.
